# Mechanical and Thermomechanical Response of Co-Cured QFRP Sandwich Structures with Additively Manufactured Fibre-Reinforced Gyroid Cores

**DOI:** 10.3390/polym18202603

**Published:** 2026-10-21

**Authors:** Vlad Buga, Teodor Adrian Badea, Madalina-Andreea Mustareata, Alexandru Ciubotariu, Raluca Maier, Andrei Timonia, Laurentiu Petre, Ciprian Ionuț Morăraș, Viorel Goanță

**Affiliations:** 1 Composite Materials Laboratory for Aeronautical Field, Romanian Research & Development Institute for Gas Turbines—COMOTI, 220D Iuliu Maniu Av., 061126 Bucharest, Romaniateodor.badea@comoti.ro (T.A.B.); madalina.mustareata@comoti.ro (M.-A.M.); raluca.maier@comoti.ro (R.M.); andrei.timonia@comoti.ro (A.T.); laurentiu.petre@comoti.ro (L.P.); 2 Mechanical Engineering, Mechatronics and Robotics Department, Mechanical Engineering Faculty, “Gheorghe Asachi” Technical University of Iasi, 700050 Iasi, Romania; ciprian-ionut.moraras@academic.tuiasi.ro (C.I.M.); viorel.goanta@academic.tuiasi.ro (V.G.)

**Keywords:** sandwich composites, QFRP, additively manufactured core, flatwise compression, dynamic mechanical analysis

## Abstract

This study investigates the mechanical and thermomechanical response of quartz-fibre-reinforced polymer (QFRP) sandwich structures incorporating additively manufactured gyroid cores. Three core variants were considered under the same nominal gyroid topology and 28% infill setting: chopped-carbon-fibre-reinforced polyphthalamide (PPA-CF), chopped-carbon-fibre-reinforced ONYX, and ONYX incorporating additional continuous carbon-fibre reinforcement (ONYX-R). Identical QFRP face sheets manufactured from woven quartz-fibre/cyanate-ester prepreg were co-cured directly onto the printed cores without a separate adhesive film. The resulting sandwich structures were characterized by flatwise compression, three-point bending, and dynamic mechanical analysis (DMA). Under flatwise compression, QFRP-ONYX-R exhibited the highest yield strength, 12.45 ± 0.16 MPa, followed by QFRP-PPA-CF at 11.70 ± 0.10 MPa, whereas QFRP-ONYX yielded at 2.00 ± 0.09 MPa. QFRP-PPA-CF exhibited the highest nominal maximum bending stress (131.55 ± 3.50 MPa) and apparent bending modulus (7.32 ± 0.07 GPa), while QFRP-ONYX-R showed an intermediate flexural response and QFRP-ONYX exhibited the greatest deformation capacity before break. DMA revealed a strong dependence of the apparent structural response on core configuration. QFRP-PPA-CF exhibited the highest storage modulus, approximately 8.4 GPa at 30 °C, and retained approximately 1.9–2.0 GPa at 160 °C. QFRP-ONYX-R displayed intermediate stiffness but the highest principal tan δ response, with peaks of 0.135–0.150 at approximately 97–100 °C, whereas QFRP-ONYX exhibited the lowest storage and loss moduli. Overall, the results show that, under a nominally similar gyroid architecture, core material and reinforcement strategy strongly modify compressive yielding, bending behaviour, apparent thermomechanical stiffness, and damping in QFRP-faced sandwich structures.

## 1. Introduction

Sandwich composites are widely used in lightweight structural applications because they can provide high bending stiffness and load-bearing capability at relatively low structural mass [1]. Their mechanical response results from the complementary roles of the face sheets and the core: the face sheets primarily sustain in-plane tensile and compressive stresses generated during bending, whereas the core maintains the separation between the skins, transfers transverse shear loads, increases the second moment of area of the structure, and contributes to overall structural stability [1,2,3]. Furthermore, depending on its material properties and internal architecture, the core can provide resistance against local face-sheet buckling, support through-thickness compressive loads, absorb impact energy, and contribute to vibration attenuation. The integrity of the skin–core interface is also essential for efficient load transfer and resistance to interfacial damage. The resulting combination provides mechanical characteristics and stiffness-to-mass ratios that are difficult to achieve using homogeneous structures of comparable mass [1,4]. Conventional sandwich cores, including honeycomb structures, polymeric foams, and other lightweight cellular materials, have therefore been extensively implemented in aerospace and other advanced engineering applications [1,4]. Nevertheless, conventional core geometries and manufacturing routes can restrict the extent to which the internal architecture is tailored to specific loading conditions or multifunctional requirements.

Additive manufacturing provides an alternative approach in which the internal architecture of a sandwich core can be defined directly during the manufacturing process. Instead of relying on a predefined cellular morphology, the designer can modify parameters such as relative density, wall thickness, unit-cell dimensions, and topology to obtain a desired structural response [5]. This architecture-driven approach is particularly relevant for polymeric cores, which can be manufactured using extrusion-based techniques with relatively high geometric freedom. The incorporation of reinforcement within the printed material can further extend the achievable mechanical performance and dimensional stability of the resulting structures [6]. Consequently, additively manufactured cores have attracted increasing interest as a means of developing sandwich structures with purpose-specific stiffness, strength, deformation and energy absorption characteristics.

Triply periodic minimal surface (TPMS) architectures have received increasing attention as lightweight cellular structures because their smooth and continuous geometries provide interconnected three-dimensional load-transfer paths without the sharp nodal intersections characteristic of many conventional strut-based lattices [7,8]. Among TPMS topologies, the gyroid is particularly attractive because of its continuous surface morphology, geometric periodicity, and high degree of architectural tunability [8]. Parameters such as relative density, wall thickness, unit-cell size, and cell orientation can be modified to tailor stiffness, strength, deformation behaviour, and energy absorption without changing the fundamental topology [8]. The geometric complexity of gyroid structures makes conventional manufacturing difficult, whereas additive manufacturing enables these architectures to be produced directly from digital models [8,9]. These characteristics have resulted in increasing interest in gyroid architectures for lightweight cellular components and sandwich cores.

The behaviour of a gyroid-core sandwich, however, cannot be considered independently of the face sheets and the interface connecting the two components. The core architecture determines how transverse loads are transferred and how deformation will develop, while the face sheets carry most of the bending and in-plane loads. Consequently, the stiffness and strength of the skins, along with the skin-core interface quality, directly influence both the structural capacity and the failure modes [10]. Experimental studies on additively manufactured gyroid sandwich structures show that core density, topology, face-sheet configuration, and interface conditions strongly influence flexural and compression performance [10].

Recent studies have progressively extended TPMS research from isolated cellular specimens toward complete sandwich structures. Junaedi et al. investigated CFRP-faced sandwich panels containing additively manufactured PLA gyroid cores with different relative densities and demonstrated that increasing core density improved core-shear and compression-related properties [10]. Stepinac et al. subsequently investigated the integration of additively manufactured gyroid building blocks into composite sandwich structural systems, further demonstrating the potential of TPMS architectures for load-bearing applications [11]. More recently, Wu et al. studied gyroid-TPMS sandwich structures manufactured from short-carbon-fibre-reinforced nylon and showed that both core relative density and face-sheet thickness strongly affect flexural stiffness, peak load, and failure mode [12]. In parallel, continuous-carbon-fibre additive manufacturing has been shown to improve the compression response of printed lattice sandwich structures, while also introducing a strong dependence on fibre path and reinforcement architecture [6]. These studies demonstrate that both material selection and cellular architecture must be considered when interpreting the performance of additively manufactured sandwich cores.

For applications in which low electromagnetic interference is required, quartz-fibre-reinforced polymer (QFRP) is a relevant face-sheet material because quartz fibres combined with suitable polymer matrices can provide a favourable combination of structural performance, thermal stability, and dielectric properties. Quartz-fibre/cyanate-ester composites have consequently been investigated for radome applications, where mechanical integrity must be combined with low electromagnetic transmission loss [13,14]. However, the electromagnetic behaviour of a complete sandwich structure depends not only on the face sheets but also on the core material and the overall multilayer architecture. Because the cores investigated in the present study contain carbon-fibre reinforcement, electromagnetic transparency of the complete sandwich configurations was not evaluated and is outside the scope of this work.

Despite the increasing interest in additively manufactured cellular structures, several research gaps remain regarding the integration of fibre-reinforced thermoplastic gyroid cores into composite sandwich configurations. Previous investigations have primarily focused on the influence of core topology, relative density, and face-sheet thickness on the mechanical performance of sandwich structures, frequently employing conventional polymeric cores or CFRP face sheets [10,11,12]. Although continuous carbon-fibre reinforcement has demonstrated considerable potential for improving the mechanical response of additively manufactured lattice structures [6], comparative experimental investigations addressing different thermoplastic matrices and reinforcement strategies within QFRP-faced sandwich structures remain limited. Furthermore, quartz-fibre/cyanate-ester composites have been predominantly investigated for applications requiring favourable mechanical, thermal, and dielectric characteristics [13,14], while their integration with additively manufactured fibre-reinforced gyroid cores has received comparatively less attention.

Another aspect that remains insufficiently documented is the combined mechanical and thermomechanical response of such hybrid sandwich configurations manufactured through direct co-curing, without an additional adhesive film. In particular, limited experimental data are available regarding how the selection of the thermoplastic core material and the incorporation of continuous carbon-fibre reinforcement influence compressive yielding, flexural stiffness and strength, deformation capacity, and temperature-dependent viscoelastic behaviour under comparable nominal gyroid geometries. Investigating these characteristics together is important for understanding the relationship between core composition, reinforcement strategy, and the overall structural performance of QFRP-faced sandwich composites.

To address these gaps, the present study investigates three QFRP sandwich configurations incorporating additively manufactured gyroid cores based on chopped-carbon-fibre-reinforced polyphthalamide (PPA-CF), chopped-carbon-fibre-reinforced ONYX, and ONYX with additional continuous carbon-fibre reinforcement (ONYX-R). All configurations employ identical quartz-fibre/cyanate-ester face sheets, directly co-cured onto the printed cores without a separate adhesive film, and a nominal gyroid infill setting of 28%. The experimental investigation combines flatwise compression, three-point bending, and dynamic mechanical analysis (DMA) to evaluate the influence of core material and reinforcement strategy on structural stiffness, strength, deformation behaviour, and temperature-dependent mechanical response. By systematically comparing these configurations, the study provides experimental evidence of the mechanical and thermomechanical trade-offs associated with different fibre-reinforced thermoplastic gyroid cores and contributes to the development of lightweight composite sandwich structures with tailored structural performance.

## 2. Materials Used and Testing Methods

### 2.1. Materials

The selection of the core materials was driven by the increasing demand for lightweight sandwich structures with enhanced mechanical performance, improved design flexibility, and simplified manufacturing for aerospace and other weight-sensitive engineering applications. Unlike conventional honeycomb cores, additively manufactured lattice structures offer the possibility of creating tailored geometries that are able to sustain specific load scenarios that would be impossible or at the very least very difficult to replicate with other manufacturing techniques.

**Figure 1:**
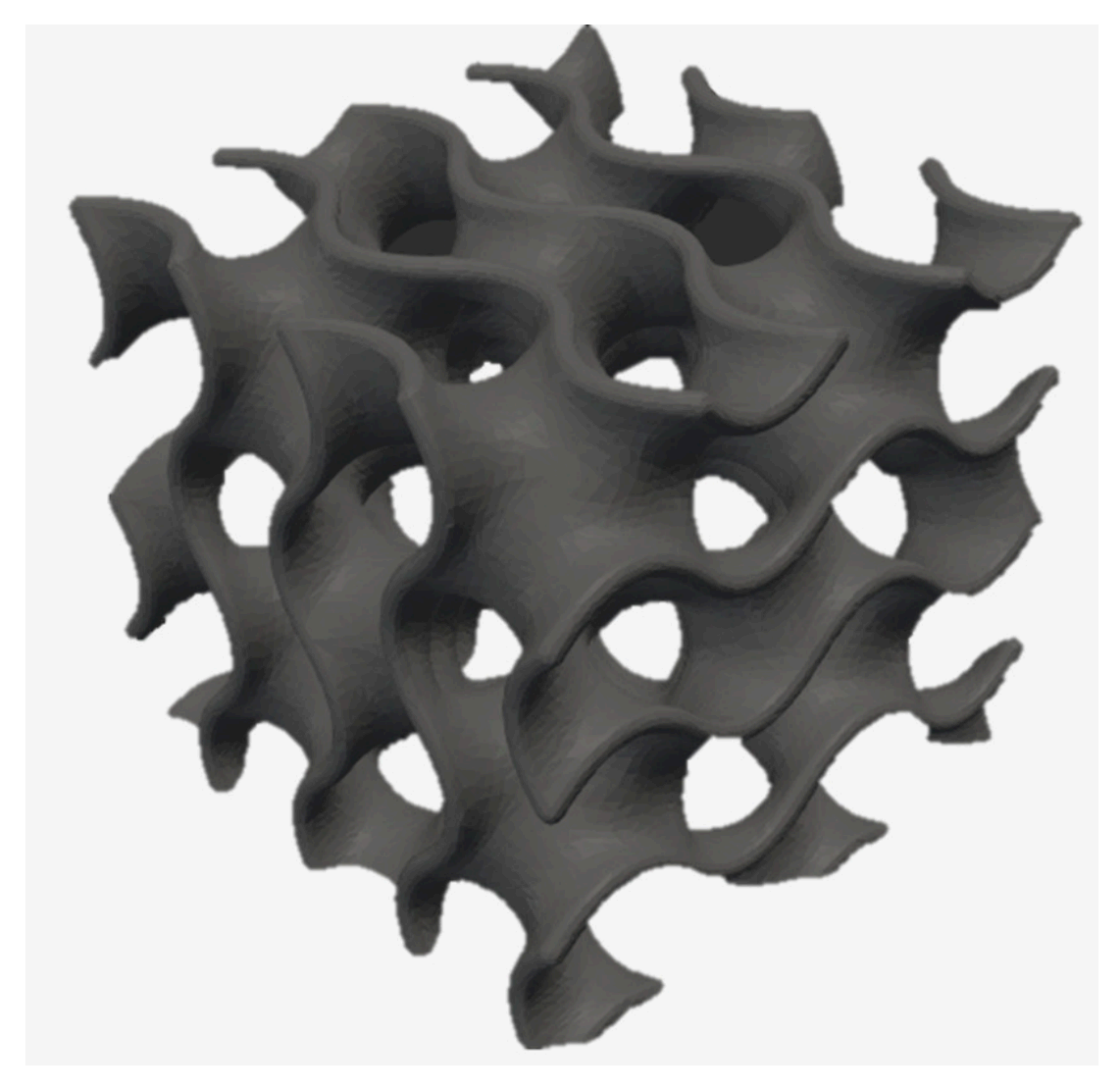
Representative visualization of the internal gyroid infill architecture employed in the additively manufactured sandwich cores, generated using a nominal infill setting of 28%.

Three additively manufactured core variants were investigated: carbon-fibre-filled polyphthalamide (PPA-CF), ONYX, and continuously reinforced ONYX (ONYX-R). ONYX is a proprietary carbon-fibre-filled polyamide-based thermoplastic filament developed by Markforged(Watertown, MA, USA), containing dispersed short carbon fibres within the polymer matrix. In the present study, ONYX-R refers to the same ONYX-based material additionally reinforced with continuous carbon fibres deposited during the additive manufacturing process. All cores had a nominal thickness of 6 mm and were produced using a nominal gyroid infill of 28%. The 28% gyroid infill represents the nominal infill density specified in the slicing software during additive manufacturing. This parameter controls the amount of deposited material within the internal gyroid architecture and should not be interpreted as an experimentally measured relative density. The same nominal infill setting was maintained for all three core configurations to provide a consistent geometric design parameter for the comparative investigation. The selected infill level was intended to provide a compromise between material consumption, structural support, and the preservation of a lightweight cellular architecture. The gyroid core geometry can be seen in Figure 1. However, the present study did not investigate different infill densities; therefore, the selected value should not be regarded as an optimized configuration.

The same nominal topology and overall core thickness were maintained to enable a comparative assessment; however, the PPA-CF and ONYX-based cores were manufactured using different printing platforms, slicing software, and layer heights.

The PPA-CF cores were manufactured on a Prusa Core One L machine(Prusa Research a.s., Prague, Czech Republic) with a 0.4 mm nozzle, using filament with a diameter of 1.75 mm, supplied by Raise3D (Raise3D Technologies, Irvine, CA, USA). The other two configurations were produced on a Markforged X7 machine (Markforged, Watertown, MA, USA). Both cores were produced using the ONYX filament (Markforged, Watertown, MA, USA); however, one was produced using the standard FDM process, while the other used the X7′s proprietary feature, in the form of continuous carbon fibre reinforcement. The ONYX material was selected because of its overall high stiffness and strength properties, whereas PPA-CF was chosen for its excellent thermomechanical stability and load-bearing capability.

The main mechanical properties of the constituent materials employed in the fabrication of the investigated sandwich structures are summarized in Table 1. The reported values are based on manufacturer technical data and are provided for material characterization purposes. It should be noted that the mechanical response of the additively manufactured gyroid cores also depends on the printing parameters, cellular architecture, and reinforcement distribution, and therefore cannot be directly inferred from the properties of the bulk constituent materials.

For the fabrication of the sandwich skins, the Astroquartz R III 4581 (AQIII) (with a nominal areal weight of 300 g/m^2^ and an 8HSatin woven) Quartz fibre with cyanate ester with an Toray EX-1515 40%wt.) resin system (Toray Advanced Composites, Morgan Hill, CA, USA) was utilized. This quartz prepreg system was chosen specifically due to its overall excellent mechanical properties, as well as its dielectric, RF transparency and high temperature stability. Since all investigated sandwich configurations employed identical QFRP face sheets, the influence of the core material on the mechanical behaviour of the sandwich structure could be directly assessed.

The sandwich panels were manufactured through a one-step co-curing process, in which the prepreg face sheets were directly bonded to the 3D printed lattice core during curing without the use of an additional adhesive film. This approach simplifies the manufacturing process while ensuring a continuous bond between the QFRP skins and the thermoplastic lattice core.

Within the slicing stage, important parameters such as layer height, speed, temperature, perimeter and top/bottom surface count and infill density were set, and in the case of the continuously reinforced ONYX core, the fibre stripe count per height. In Table 2 the parameters utilized for manufacturing the three types of cores are presented, along with the machine they were manufactured on.

The estimated continuous-carbon-fibre volume fraction of the ONYX-R configuration varied between the specimen sets used for compression, bending, and DMA.

Following the fabrication of the gyroid lattice cores, the sandwich specimens were assembled by manually applying QFRP prepreg face sheets to both sides of each printed core. The prepreg plies were previously cut to the required dimensions using a CNC Eastman Eagle S125-60x8 cutting system (Eastman Machine Company, Buffalo, NY, USA) according to the dimensions required by each test specimen.

**Figure 2:**
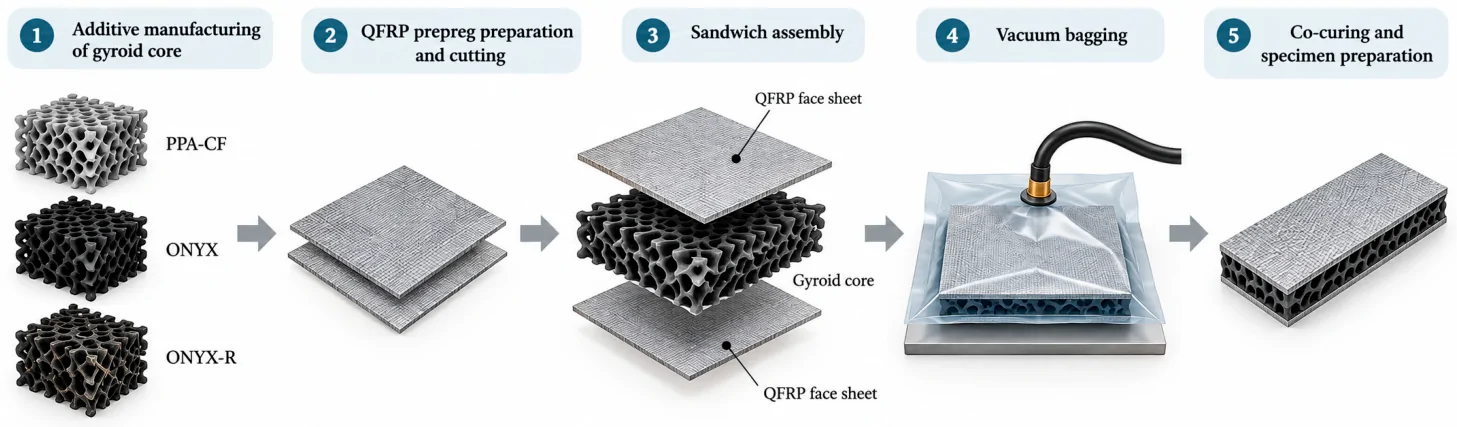
Schematic representation of the manufacturing process of QFRP sandwich structures incorporating additively manufactured fibre-reinforced gyroid cores: (1) additive manufacturing of PPA-CF, ONYX, and ONYX-R cores; (2) preparation and CNC cutting of QFRP prepreg plies; (3) sandwich assembly; (4) vacuum bagging; and (5) co-curing and final specimen preparation.

A symmetric laminate configuration was produced for each sandwich specimen by placing two QFRP prepreg plies on either side of a 3D-printed gyroid core, for a total of four plies per sample. Manual alignment was performed to ensure full coverage of the core and correct orientation of the composite face sheets. Rather than utilizing a separate adhesive film, adhesion at the QFRP—thermoplastic core interface was formed naturally during the co-curing process. The main manufacturing stages of the investigated QFRP sandwich structures, from the additive manufacturing of the gyroid cores to the final specimen preparation, are schematically illustrated in Figure 2.

After assembly, the sandwich specimens were constrained between flat metallic plates to maintain dimensional stability and promote uniform contact between the QFRP face sheets and the printed core. The complete assembly was enclosed in a vacuum bag using the required release and breather materials. A vacuum level of −0.9 bar was applied and maintained throughout the curing cycle. The specimens were heated to 120 °C at a rate of 3 °C/min, held for 120 min, and subsequently cooled at approximately 3 °C/min. No separate structural adhesive film was introduced; bonding at the QFRP–core interface resulted from the prepreg resin during the co-curing cycle.

### 2.2. Experimental Setup

**Figure 3:**
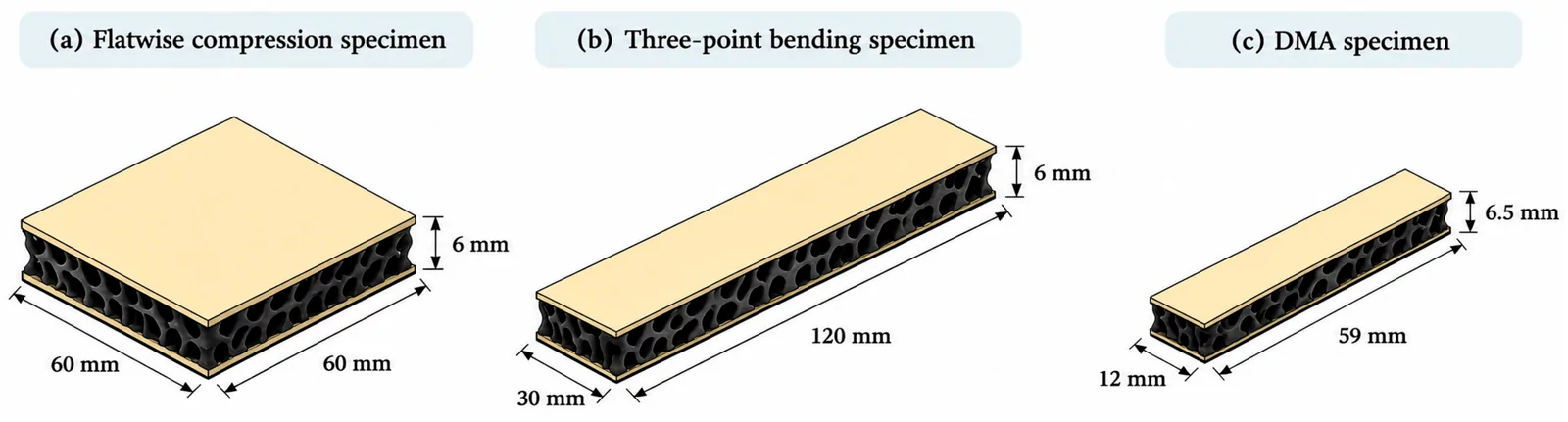
Schematic representation of the QFRP sandwich specimen geometries used for (**a**) flatwise compression, (**b**) three-point bending, and (**c**) dynamic mechanical analysis (DMA). Dimensions are expressed in millimetres. Drawings are not to scale.

The investigated QFRP sandwich structures were prepared in different specimen geometries according to the requirements of each experimental procedure. Flatwise compression specimens had in-plane dimensions of 60 × 60 mm, while three-point bending specimens measured 120 × 30 mm. For dynamic mechanical analysis (DMA), specimens with nominal dimensions of 59 × 12 × 6.5 mm were employed. All specimens incorporated additively manufactured gyroid cores enclosed between QFRP face sheets. A schematic representation of the specimen geometries, including their relevant dimensions, is presented in Figure 3.

#### 2.2.1. Compression Tests

The compressive mechanical response of the sandwich composite structures was characterized in accordance with ASTM C365 [15] using an Instron 8801 universal testing machine (Instron, Norwood, MA, USA)equipped with a 100 kN load cell. All tests were conducted under controlled laboratory conditions of 23 ± 3 °C and 50 ± 5% relative humidity. Compression was applied under displacement-controlled loading at a constant crosshead rate of 0.5 mm min^−1^ until specimen failure. Square specimens with in-plane dimensions of 60 × 60 mm, corresponding to a nominal loaded area of 3600 mm^2^, were tested through the full thickness of the sandwich construction. A minimum of three replicate specimens was tested for each sandwich configuration to evaluate the reproducibility of the measured mechanical response.

#### 2.2.2. 3-Point Bending Tests

The core shear response of the sandwich structures was characterized by three-point bending in accordance with ASTM C393/C393M [16]. This test method was used to evaluate the resistance of the sandwich panels to core shear and associated compressive failure modes. Mechanical testing was performed using an Instron 34SC-5 universal testing machine (Instron, ITW—Illinois Tool Works, Norwood, MA, USA) equipped with a 5 kN load cell. Rectangular specimens with dimensions of 120 × 30 mm were tested under controlled laboratory conditions of 23 ± 3 °C and 50 ± 5% relative humidity. Each specimen was centrally positioned on a three-point bending fixture with a support span of 70 mm. Cylindrical loading and support rollers with a radius of 5 mm were employed. The load was applied under displacement-controlled conditions at a constant crosshead rate of 6 mm min^−1^ until specimen failure or until the mid-span deflection reached a magnitude equal to the total sandwich thickness, whichever occurred first.

#### 2.2.3. Dynamic Mechanical Analyzer (DMA)

Dynamic mechanical analysis (DMA) was conducted using a TA Instruments DMA 850 analyser (New Castle, DE, USA) operated in three-point bending mode. The testing procedure was in accordance with ASTM D7028-07(2024) [17]. Measurements were performed over a temperature range extending from ambient temperature to 160 °C, using a heating rate of 5 ± 1 °C min^−1^ and an oscillation frequency of 1 Hz. For each sandwich configuration, three replicate rectangular specimens with nominal dimensions of 59 ± 1 mm × 12 ± 1 mm × 6.5 ± 0.5 mm were tested. The nominal thickness of the additively manufactured gyroid cores was 6 mm, whereas the overall thickness of the sandwich specimens was approximately 6.5 mm, including the QFRP face sheets.

#### 2.2.4. Experimental Repeatability and Data Processing

To assess the repeatability of the experimental results, three specimens were evaluated for each sandwich configuration under the investigated testing conditions. The specimens were manufactured using the nominally identical core architecture, face-sheet layup, and co-curing procedure described in Section 2.1. The mechanical tests were performed using consistent specimen geometries, loading conditions, and testing equipment for each test category. The experimental results are reported as mean values accompanied by their standard deviations, allowing the variability between specimens to be assessed. For the DMA measurements, the individual experimental curves were compared to evaluate the consistency of the temperature-dependent thermomechanical response.

All configurations were initially tested using three replicate specimens. However, one QFRP-PPA-CF compression specimen was excluded from the statistical analysis, as detailed in the note accompanying Table 3. Accordingly, the reported compression statistics for this configuration are based on two specimens, whereas the remaining datasets comprise three specimens per configuration.

## 3. Results

To facilitate the comparison of the investigated sandwich configurations, the compression and three-point bending results are presented as average stress–strain curves obtained from replicate specimens. The corresponding quantitative mechanical properties are reported as mean values with standard deviations in Table 3 and Table 4, providing an assessment of the experimental variability.

### 3.1. Compression Test Results

**Figure 4:**
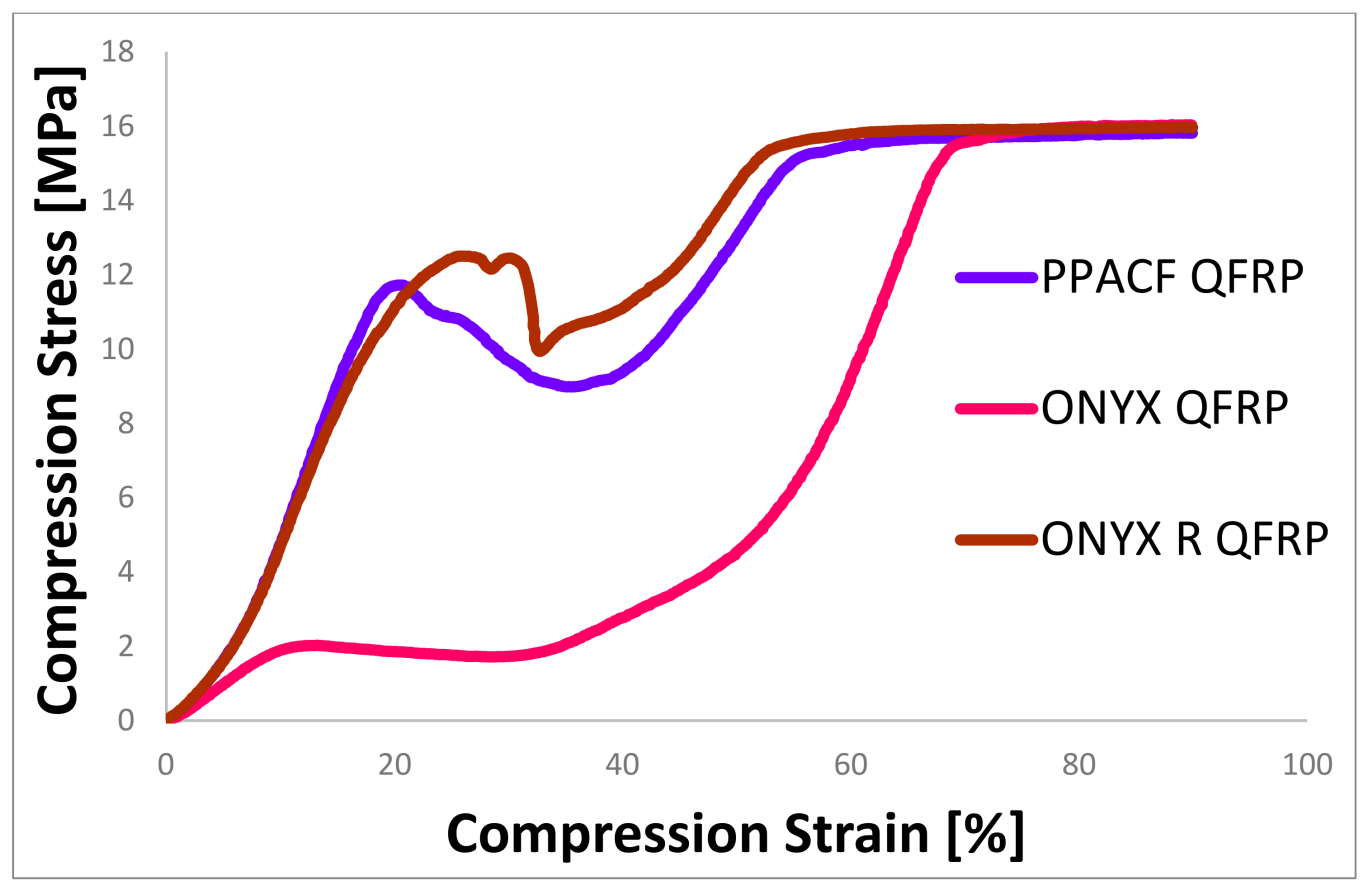
Average flatwise compressive stress–strain curves of the investigated QFRP sandwich configurations.

Figure 4 presents the flatwise compressive stress–strain curves obtained for the QFRP-PPA-CF, QFRP-ONYX-R, and QFRP-ONYX sandwich configurations. Three specimens were tested for each configuration, and the corresponding compression modulus, 0.2% offset yield strength, and compressive strength values are summarized in Table 3 as mean values with standard deviations. QFRP-PPA-CF exhibited a yield strength of 11.70 ± 0.10 MPa and a compression modulus of 86.26 ± 0.42 MPa. QFRP-ONYX-R exhibited the highest yield strength, at 12.45 ± 0.16 MPa, together with a compression modulus of 76.24 ± 2.76 MPa. The most pronounced difference was observed for QFRP-ONYX, which exhibited a substantially lower yield strength of 2.00 ± 0.09 MPa and a compression modulus of 22.08 ± 2.69 MPa.

### 3.2. Three-Point Bending Results

**Figure 5:**
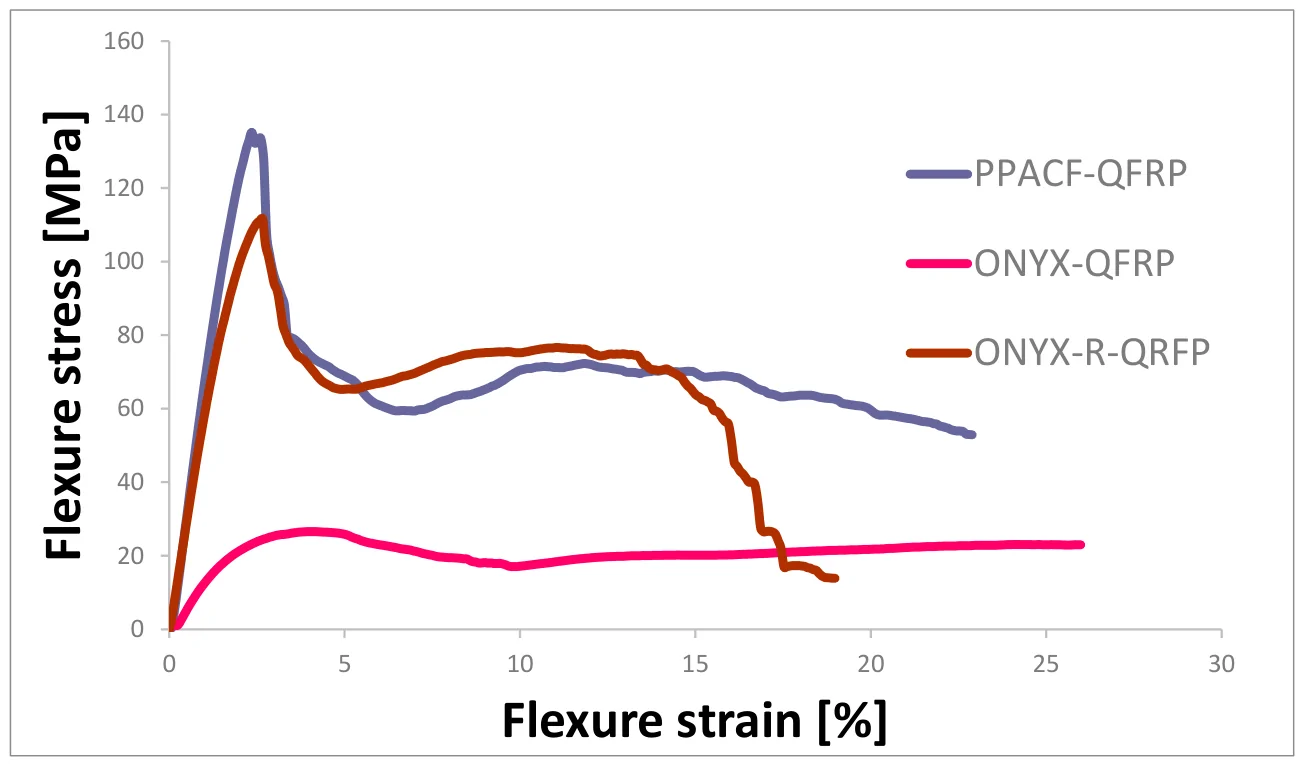
Average flexural stress–strain curves of the investigated QFRP sandwich configurations obtained from three-point bending tests.

Figure 5 presents the flexural stress–strain curves obtained for the three investigated sandwich configurations, while the corresponding experimental parameters are summarized in Table 4. The results include maximum flexural stress, flexural modulus, flexural stress and strain at break, and absorbed energy. The average stress–strain curves are presented to facilitate a direct comparison of the flexural responses of the three investigated sandwich configurations.

The QFRP-PPA-CF specimens exhibited a maximum flexural stress of 131.55 ± 3.50 MPa and a flexural modulus of 7319 ± 72 MPa. Their stress–strain curves were characterized by a steep initial linear response followed by a distinct maximum and an abrupt post-peak stress reduction. At break, the specimens retained a flexural stress of 125.49 ± 2.18 MPa at a corresponding flexural strain of 2.63 ± 0.10%. The energy absorbed up to break was 3.18 ± 0.29 J. Following the initial failure event, the specimens continued to sustain residual loads, although their post-peak responses differed considerably between specimens.

QFRP-ONYX-R exhibited a maximum flexural stress of 109.62 ± 3.00 MPa, approximately 17% lower than that of QFRP-PPA-CF. The flexural modulus was 5977 ± 93 MPa. At break, the specimens retained a flexural stress of 108.76 ± 3.21 MPa, corresponding to a flexural strain of 2.68 ± 0.20%. The absorbed energy up to break was 2.75 ± 0.33 J. The three specimens exhibited comparable flexural responses, with failure occurring close to the maximum load, followed by a pronounced post-peak reduction in stress. The strain at break was substantially lower than that recorded for QFRP-ONYX (9.38 ± 1.02%), while the maximum flexural stress was considerably higher.

QFRP-ONYX exhibited a maximum flexural stress of 25.91 ± 1.59 MPa and a flexural modulus of 1483 ± 67 MPa, both markedly lower than those measured for the QFRP-PPA-CF and QFRP-ONYX-R configurations. The flexural stress increased progressively and reached its maximum at approximately 4.0–4.1% strain. Beyond the peak, the stress decreased moderately and then remained relatively stable. At break, the specimens showed a flexural stress of 19.62 ± 2.01 MPa and a flexural strain of 9.38 ± 1.02%. The energy absorbed up to break was 3.13 ± 0.48 J. Overall, the QFRP-ONYX configuration exhibited a more progressive deformation and failure response, without the pronounced initial stress drop observed for QFRP-PPA-CF and QFRP-ONYX-R.

### 3.3. DMA Measurements

**Figure 6:**
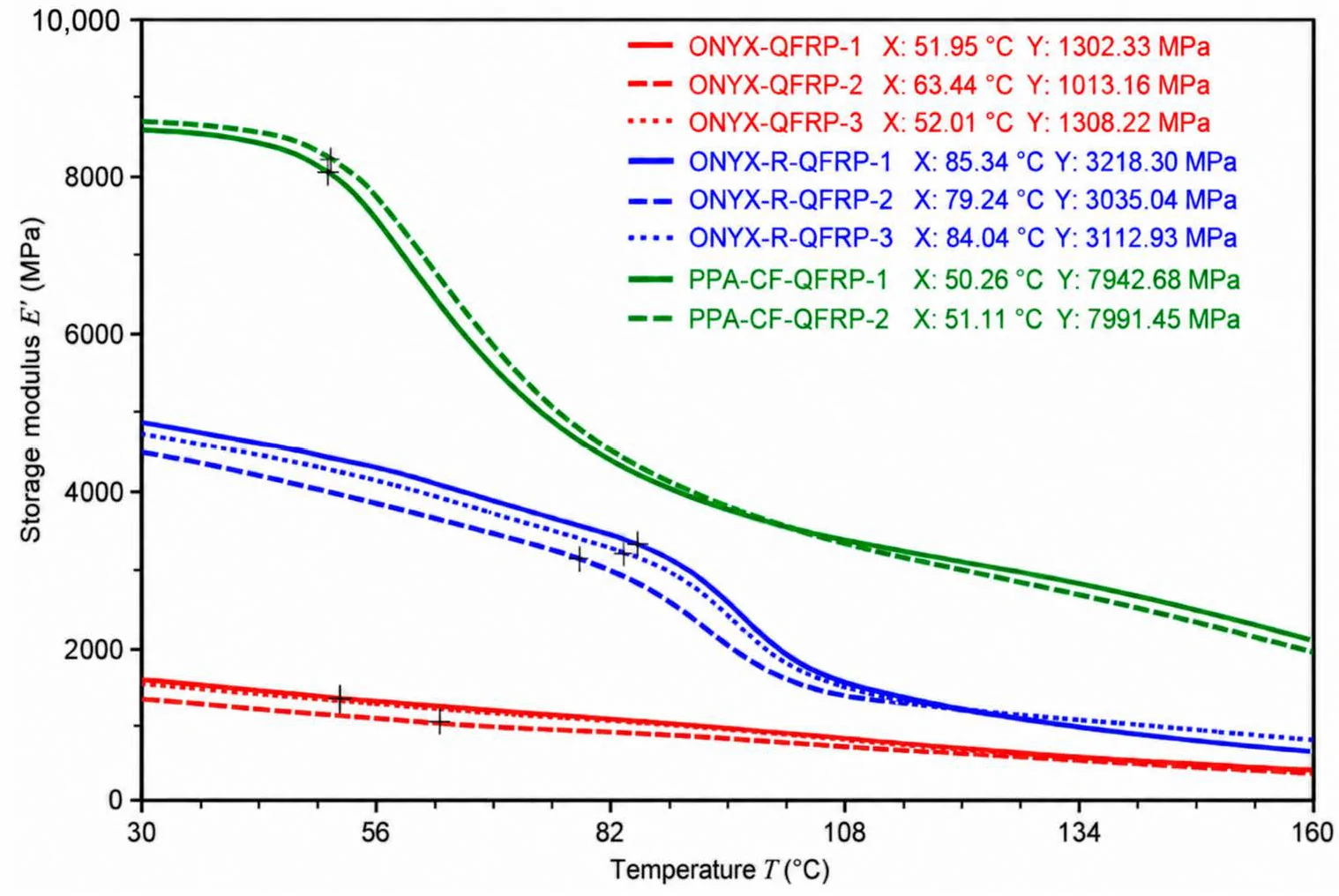
Apparent storage modulus (E′) as a function of temperature for the investigated sandwich configurations.

Figure 6 shows the variation in the storage modulus (E′) with temperature for the investigated QFRP sandwich structures. The storage modulus showed a pronounced dependence on the 3D-printed core material. At 30 °C, the QFRP-PPA-CF specimens exhibited the highest stiffness, with E′ values of approximately 8.4 GPa, followed by QFRP-ONYX-R at approximately 4.3–4.7 GPa and QFRP-ONYX at approximately 1.3–1.6 GPa.

All configurations underwent progressive stiffness loss with increasing temperature. QFRP-PPA-CF showed a pronounced modulus reduction beginning at approximately 50–55 °C, but maintained the highest absolute E′, retaining approximately 3.2–3.4 GPa at 110 °C and 1.9–2.0 GPa at 160 °C. The continuously reinforced ONYX core configuration exhibited a moderate reduction up to 80–85 °C (≈3.0–3.2 GPa), followed by a more pronounced drop between 90 and 105 °C, reaching about 0.6–0.7 GPa at 160 °C. In contrast, the ONYX core showed the lowest storage modulus (≈1.0–1.3 GPa) and a continuous decline throughout the test to approximately 0.3–0.4 GPa at 160 °C.

**Figure 7:**
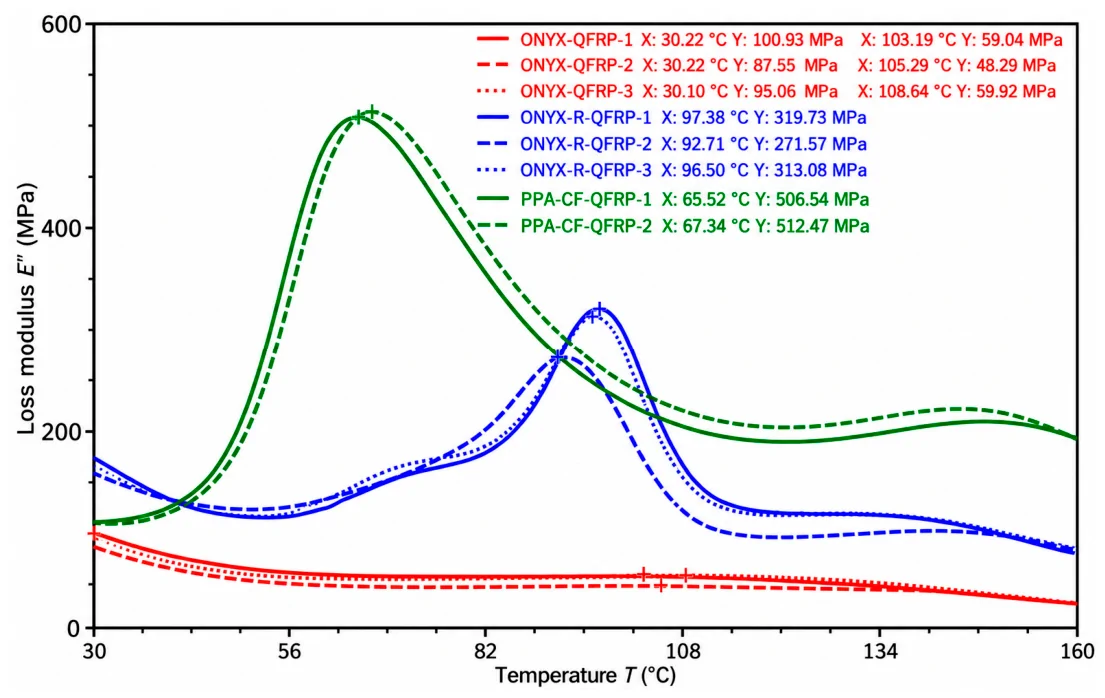
Apparent loss modulus (E″) as a function of temperature for the investigated sandwich configurations.

Figure 7 shows the variation in the loss modulus (E″) with temperature for the investigated QFRP sandwich structures.

QFRP-PPA-CF produced the highest and broadest primary peaks, with maximum E″ values of 506.5 and 512.5 MPa at 65.5 and 67.3 °C, respectively. After the maximum, E″ decreased gradually but remained comparatively high, at approximately 190–220 MPa up to 160 °C. A weak secondary increase was observed above approximately 130 °C.

QFRP-ONYX-R exhibited narrower peaks at 92.7–97.4 °C, with maximum E″ values ranging from 271.6 to 319.7 MPa. Following these peaks, the loss modulus decreased sharply, although a weak high-temperature contribution remained visible. By comparison, QFRP-ONYX presented substantially lower E″ values. Following an initial decrease, broad and poorly defined maxima of approximately 48.3–59.9 MPa occurred between 103.2 and 108.6 °C. The replicate curves showed good agreement in peak position and overall profile.

**Figure 8:**
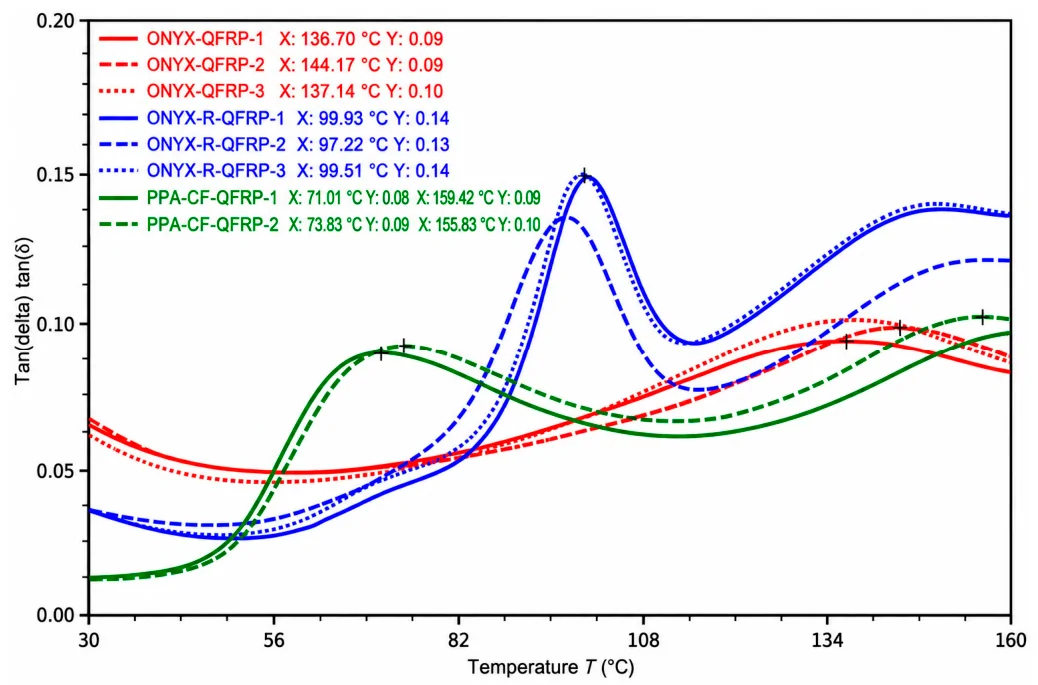
Damping factor (tan δ) as a function of temperature for the investigated sandwich configurations.

Figure 8 presents the variation in the damping factor (tan δ) with temperature for the investigated QFRP sandwich configurations, highlighting the differences in peak position and damping response among the three core materials. Based on the tan δ maxima, the principal apparent relaxation regions occurred at approximately 71–74 °C for QFRP-PPA-CF, 97–100 °C for QFRP-ONYX-R, and 137–144 °C for QFRP-ONYX.

The measured E′, E″, and tan δ responses demonstrate distinct temperature-dependent behaviours among the investigated sandwich configurations, with differences in initial stiffness, modulus retention, and the positions of the principal damping peaks.

For the DMA measurements, the individual experimental curves were retained to illustrate the temperature-dependent response and the variability among replicate specimens. Unlike the compression and three-point bending results, the DMA responses are presented as individual measurements rather than averaged curves, allowing differences in storage modulus, loss modulus, and damping behaviour to be observed directly.

## 4. Discussion

### 4.1. Compression Test Outcome

The mechanical variations observed during flatwise compression reflect the combined influence of the core material, cellular geometry, face-sheet properties, and face sheet–core interfacial integrity. A pronounced difference in compressive stiffness was observed between QFRP-ONYX and QFRP-ONYX-R. The compression modulus increased from 22.08 ± 2.69 MPa for QFRP-ONYX to 76.24 ± 2.76 MPa for QFRP-ONYX-R, corresponding to an approximately 3.5-fold increase. This substantial increase indicates the contribution of the continuous carbon-fibre reinforcement to the stiffness of the ONYX-based core. QFRP-PPA-CF exhibited the highest compression modulus, reaching 86.26 ± 0.42 MPa despite the absence of continuous-fibre reinforcement.

The influence of the core configuration was also pronounced in the yielding response. QFRP-ONYX-R exhibited the highest 0.2% offset yield strength, at 12.45 ± 0.16 MPa, followed closely by QFRP-PPA-CF at 11.70 ± 0.10 MPa. In contrast, QFRP-ONYX yielded at only 2.00 ± 0.09 MPa, confirming a markedly more compliant response before the onset of yielding.

**Figure 9:**
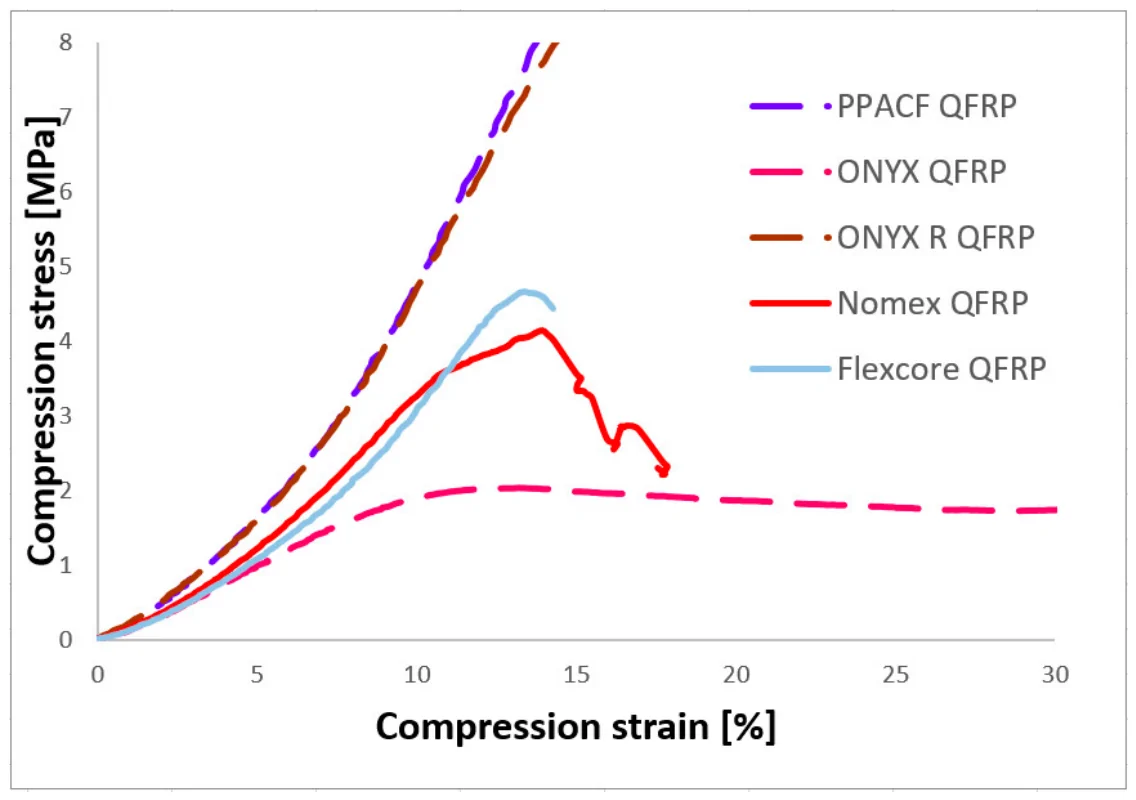
Conventional aramide core sandwich structures compared to QFRP—3D printed gyroid core sandwiches.

For comparison, conventional QFRP sandwich structures incorporating Nomex honeycomb and Flexcore cores were previously investigated in [18], with reported yield stresses of approximately 4 MPa and 4.5 MPa, respectively. The yield strengths measured for QFRP-PPA-CF and QFRP-ONYX-R were therefore substantially higher, exceeding those of the conventional sandwich structures by at least 7.2 MPa. QFRP-ONYX represented a clear exception, with a yield strength of approximately 2 MPa, below the values reported for both conventional core systems. Its comparatively low yield strength is consistent with the more compliant response of the polyamide-based core [19]. These differences in compressive behaviour are illustrated in Figure 9.

The improved compressive yielding of the additively manufactured cores is important to consider in relation to the associated mass penalty. The QFRP-PPA-CF and QFRP-ONYX-R compression specimens had average masses of 15.59 ± 0.07 g and 17.55 ± 0.04 g, respectively, compared with 9.35 ± 0.04 g for Nomex-QFRP and 9.33 ± 0.16 g for Flexcore-QFRP. Thus, PPA-CF and ONYX-R were approximately 67% and 88% heavier than the conventional sandwich configurations. However, this increase in mass was accompanied by a disproportionately larger improvement in yield strength. QFRP-PPA-CF and QFRP-ONYX-R reached yield strengths of 11.70 ± 0.10 MPa and 12.45 ± 0.16 MPa, respectively, compared with approximately 4 MPa for Nomex-QFRP and 4.5 MPa for Flexcore-QFRP. When normalized by specimen mass, QFRP-PPA-CF and QFRP-ONYX-R retained higher yield-performance indices of approximately 0.75 and 0.71 MPa/g, respectively, compared with approximately 0.43 MPa/g for Nomex-QFRP and 0.48 MPa/g for Flexcore-QFRP. In contrast, QFRP-ONYX combined a higher specimen mass (14.39 ± 0.06 g) with a substantially lower yield strength (2.00 ± 0.09 MPa), resulting in the lowest mass-normalized performance. These results indicate that the mass penalty associated with the printed gyroid cores can be mechanically justified for PPA-CF and continuously reinforced ONYX, but not for the non-continuously reinforced ONYX configuration.

The substantially lower compressive yield strength and modulus of QFRP-ONYX can be attributed to differences in the mechanical response of the core materials and their reinforcement strategies. Although all configurations employed the same nominal gyroid infill setting, the ONYX core contained only dispersed short carbon fibres, whereas ONYX-R incorporated additional continuous carbon-fibre reinforcement. The latter may improve load transfer within the printed structure and restrict local deformation of the cellular walls under compressive loading. In contrast, the lower stiffness of the unreinforced-by-continuous-fibre ONYX architecture may promote earlier local deformation and instability, resulting in a lower apparent compressive modulus and earlier yield. The higher compressive stiffness of QFRP-PPA-CF may also be associated with the properties of its carbon-fibre-filled polyphthalamide matrix. Nevertheless, differences in printing parameters and the resulting internal morphology may contribute to the observed behaviour, and the individual effects of these factors cannot be isolated from the present experimental results.

### 4.2. Three-Point Bending Test Outcome

Since all specimens had identical QFRP skins and comparable sandwich geometry, the observed differences in flexural behaviour were primarily associated with the core configuration. In sandwich bending, the face sheets carry the principal tensile and compressive stresses, while the core maintains their separation and transfers transverse shear between them. Because the PPA-CF and ONYX cores were manufactured on different platforms using different processing settings, the observed differences should be attributed to the tested core configurations as a whole and not to material composition alone.

The higher maximum flexural stress and modulus of QFRP-PPA-CF suggest that its core provided greater resistance to shear deformation and more effective load transfer between the face sheets. This behaviour is consistent with greater resistance of the PPA-CF cell walls to bending and shear deformation, while the carbon-fibre reinforcement likely restricted deformation of the polymer matrix and increased resistance to local instability. QFRP-PPA-CF consequently exhibited approximately 20% higher maximum flexural stress and 22% higher flexural modulus than QFRP-ONYX-R.

The abrupt post-peak stress reduction observed for both QFRP-PPA-CF and QFRP-ONYX-R suggests the occurrence of a localized damage or instability event near the maximum load. Potential mechanisms include local core crushing, cell-wall buckling, shear deformation, indentation beneath the loading roller, or partial skin–core interfacial debonding. Since the specimens retained load-bearing capacity after the initial stress reduction, this first damage event did not necessarily correspond to complete structural failure. For QFRP-PPA-CF, the comparatively high stiffness and strength may also have reduced the capacity of the core to accommodate localized deformation progressively, resulting in a more abrupt onset of damage. The subsequent differences among the post-peak curves may reflect local variations in the printed core architecture or skin–core interfacial integrity.

QFRP-ONYX-R exhibited lower stiffness and maximum flexural stress than QFRP-PPA-CF, with values of 5977 ± 93 MPa and 109.62 ± 3.00 MPa, respectively. Nevertheless, the incorporation of continuous carbon-fibre reinforcement substantially increased the structural response relative to QFRP-ONYX. Its post-peak behaviour suggests progressive core deformation rather than an immediate loss of load-bearing capacity. Possible mechanisms include progressive cell-wall bending, rotation, and localized collapse, which may contribute to stress redistribution within the core. The relatively small scatter in maximum stress, modulus, and strain at break indicates a consistent pre-failure mechanical response among the specimens. The energy absorbed up to break was 2.75 ± 0.33 J.

QFRP-ONYX exhibited a substantially more compliant response, with a flexural modulus of 1483 ± 67 MPa and a maximum flexural stress of 25.91 ± 1.59 MPa. The more compliant core underwent greater deformation and was less effective in maintaining the relative separation and alignment of the face sheets during bending. Consequently, a greater proportion of the imposed displacement was accommodated through core deformation rather than through the development of high tensile and compressive stresses in the QFRP skins. At the same time, the compliant cellular structure allowed substantially greater deformation before break, with a flexural strain at break of 9.38 ± 1.02%. Despite its much lower stress level, this greater deformation capacity resulted in an energy at break of 3.13 ± 0.48 J, comparable to the 3.18 ± 0.29 J measured for QFRP-PPA-CF and higher than the 2.75 ± 0.33 J obtained for QFRP-ONYX-R.

The pronounced differences in flexural performance can be explained by the combined influence of the core material stiffness, reinforcement strategy, and deformation behaviour of the gyroid architecture. Under three-point bending, the QFRP face sheets primarily resist tensile and compressive stresses, while the core maintains their separation and transfers transverse shear loads. Consequently, the mechanical response of the cellular core significantly influences the apparent flexural stiffness and load-bearing capacity of the sandwich structure. The higher flexural stress and modulus of QFRP-PPA-CF may be associated with the greater resistance of its carbon-fibre-filled polyphthalamide core to cellular wall deformation, whereas the additional continuous carbon-fibre reinforcement in QFRP-ONYX-R may improve load transfer and restrict local deformation within the printed architecture. In contrast, QFRP-ONYX, which contains only short carbon-fibre reinforcement, exhibited substantially lower stiffness and strength but a considerably higher strain at break. This behaviour suggests a more compliant cellular response, allowing progressive deformation under bending rather than the abrupt post-peak stress reduction observed for the other two configurations. However, the precise contribution of individual failure mechanisms, including cell-wall buckling, local crushing, and skin–core interfacial damage, cannot be established without direct examination of the fractured specimens.

Overall, QFRP-PPA-CF exhibited the highest flexural stiffness and maximum stress, whereas QFRP-ONYX showed the greatest deformation capacity but substantially lower structural resistance. QFRP-ONYX-R displayed intermediate stiffness and strength, demonstrating the substantial reinforcement effect provided by the continuous carbon fibres relative to QFRP-ONYX.

### 4.3. DMA Test Outcome

The substantially higher E′ of QFRP-PPA-CF indicates that the PPA-CF core provided the greatest resistance to elastic deformation and contributed most effectively to the global stiffness of the sandwich structure. This response is consistent with the high rigidity of the PPA-based matrix and the restriction of polymer-chain mobility by the carbon fibres. Although its modulus began to decrease at a lower temperature than that of QFRP-ONYX-R, QFRP-PPA-CF retained the greatest absolute stiffness over the entire interval. Consequently, transition onset and retained structural stiffness should be considered separately when evaluating thermal performance.

Continuous carbon fibre reinforcement considerably improved the stiffness of the ONYX core, with QFRP-ONYX-R exhibiting an initial modulus approximately three times higher than that of QFRP-ONYX. The fibres increase load-transfer efficiency and constrain deformation of the polymeric matrix. Nevertheless, the marked decrease above approximately 85–105 °C demonstrates that the response remained strongly influenced by thermally induced matrix softening. The ONYX core sandwich provided the lowest stiffness because its behaviour was governed predominantly by the comparatively compliant polymer phase.

QFRP-PPA-CF exhibited the highest and broadest loss-modulus peak because its high storage modulus allowed the structure to store more mechanical energy before part of that energy was dissipated through polymer-chain relaxation. The broad peak, together with the simultaneous decrease in E′, suggests that softening developed progressively over a relatively wide temperature range. Therefore, the high E″ should be attributed to the combination of high stiffness and viscous relaxation rather than to superior damping efficiency alone.

The QFRP-ONYX-R peak occurred at a higher temperature and was narrower, indicating that the dominant relaxation was delayed but took place over a more restricted temperature interval, followed by a rapid reduction in load-bearing capacity. QFRP-ONYX displayed only a weak and poorly defined peak, reflecting its low stiffness and limited capacity to store and dissipate mechanical energy. Overall, QFRP-PPA-CF provided the greatest absolute dissipative response, while QFRP-ONYX-R achieved the most efficient damping relative to the energy stored, as confirmed by its higher tan δ.

Since tan δ represents the ratio E″/E′, QFRP-ONYX-R demonstrated the greatest damping efficiency during its principal transition, dissipating the largest fraction of the elastically stored energy. QFRP-PPA-CF combined superior stiffness with lower relative damping, showing that its high E″ values resulted partly from the greater amount of energy stored rather than from a higher dissipated fraction.

An additional distinction should be made between the apparent flexural modulus obtained from quasi-static three-point bending and the storage modulus (E′) determined through DMA. Although both measurements involve bending deformation, they characterize the sandwich response under different loading conditions. The quasi-static flexural modulus is derived from the initial mechanical response under monotonic loading, whereas the DMA storage modulus represents the elastic component of the response under small-amplitude oscillatory loading at a frequency of 1 Hz. Consequently, the two quantities are not directly interchangeable. In the present study, QFRP-PPA-CF exhibited a flexural modulus of 7.32 GPa, compared with 5.98 GPa for QFRP-ONYX-R, while their corresponding storage moduli at approximately 30 °C were 8.4 GPa and 4.3–4.7 GPa, respectively. These differences suggest that the influence of core material and reinforcement configuration on the apparent sandwich stiffness depends on the applied loading conditions and measurement method.

The broad or multiple tan δ features indicate that the sandwich specimens did not behave as single homogeneous polymers. Contributions from the printed core, QFRP skin matrix, fibre-matrix interfaces, and core–skin interfaces may overlap. The secondary high-temperature responses should therefore not automatically be assigned to a specific constituent without DMA measurements of the individual core and skin materials. Overall, QFRP-PPA-CF offered the best stiffness and high-temperature stiffness retention, QFRP-ONYX-R provided the highest damping efficiency and a well-defined principal relaxation peak near 98 °C, and QFRP-ONYX displayed the lowest stiffness and absolute energy-dissipation capacity.

## 5. Conclusions

This study investigated QFRP-faced sandwich structures incorporating additively manufactured gyroid cores based on PPA-CF, chopped-CF ONYX, and ONYX with additional continuous carbon-fibre reinforcement. Under the nominally similar gyroid architecture considered here, the experimental results demonstrate that the core material and reinforcement strategy strongly influence the compressive, bending, and thermomechanical responses of the complete sandwich structure.

Under flatwise compression, QFRP-ONYX-R exhibited the highest yield strength (12.45 ± 0.16 MPa), closely followed by QFRP-PPA-CF (11.70 ± 0.10 MPa), whereas QFRP-ONYX showed a substantially lower yield strength of 2.00 ± 0.09 MPa. A similar trend was observed for compressive stiffness, with compression moduli of 76.24 ± 2.76 MPa for QFRP-ONYX-R and 22.08 ± 2.69 MPa for QFRP-ONYX. These results indicate that continuous carbon-fibre reinforcement had a pronounced effect on both the initial stiffness and yielding response of the ONYX-based core.

The bending response showed a different trend. QFRP-PPA-CF exhibited the highest maximum flexural stress and flexural modulus, whereas QFRP-ONYX-R showed intermediate stiffness and strength together with a progressive post-peak response. QFRP-ONYX displayed substantially lower peak resistance and stiffness but sustained considerably greater deformation before break. Despite its lower stress level, QFRP-ONYX reached an energy at break of 3.13 ± 0.48 J, comparable to that of QFRP-PPA-CF (3.18 ± 0.29 J) and higher than that of QFRP-ONYX-R (2.75 ± 0.33 J). These results indicate that higher initial stiffness and peak resistance do not necessarily correspond to greater deformation capacity or higher energy absorption up to break.

The DMA measurements further showed that QFRP-PPA-CF retained the highest apparent structural stiffness throughout the investigated temperature range. QFRP-ONYX-R exhibited lower absolute stiffness but the highest principal tan δ response, indicating greater relative damping during its dominant relaxation region. Because the DMA specimens comprise multiple polymeric constituents and interfaces, the observed tan δ maxima should be interpreted as apparent structural relaxation temperatures rather than intrinsic glass-transition temperatures of individual constituent materials.

Overall, no single-core configuration maximized all measured mechanical responses. QFRP-PPA-CF exhibited the highest flexural stiffness and maximum flexural stress, whereas continuous carbon-fibre reinforcement of the ONYX core substantially increased both compressive stiffness and yield strength compared with the non-continuously reinforced QFRP-ONYX configuration. QFRP-ONYX, despite its substantially lower stiffness and strength, exhibited the greatest deformation capacity under bending. These results demonstrate that the mechanical and thermomechanical behaviour of QFRP-faced sandwich structures can be tailored through the selection of the additively manufactured core material and reinforcement strategy. Further work should quantify the realized core density and geometry, directly characterize the skin–core interface and associated failure mechanisms, and evaluate specific mechanical properties and electromagnetic performance for multifunctional radome applications.

## Figures and Tables

**Table 1:** Manufacturer-reported mechanical properties of the constituent materials used in the investigated sandwich configurations.

Material	Properties	Source
PPA-CF (Raise3D)	Tensile Strength: 110 MPa; Tensile Modulus: 8896 MPa; Elongation at Break: 2.18%; Bending Modulus: 8487 MPa; Bending Strength: 176 MPa	Raise3D—Technical Data Sheet
ONYX (Markforged)	Tensile Strength: 40 MPa; Tensile Modulus: 2400 MPa; Elongation at Break: 25%; Bending Modulus: 3000 MPa; Bending Strength: 71 MPa	Markforged—Onyx Material Data Sheet
Continuous carbon fibre (Markforged)	Tensile Strength: 800 MPa; Tensile Modulus: 60,000 MPa; Elongation at Break: 1.5%; Bending Modulus: 51,000 MPa; Bending Strength: 540 MPa	Markforged—Carbon Fiber Material Data Sheet
QFRP (Astroquartz R III 4581/EX-1515)	Tensile Strength: 757 MPa; Tensile Modulus: 24,000 MPa; Bending Modulus: 21,800 MPa; Bending Strength: 738 MPa; Fibre Content: 55%	Toray—Astroquartz R III 4581/EX-1515 Technical Data Sheet

**Table 2:** 3D-printed core features.

Feature	Core Type				
Material	PPA-CF	ONYX	Carbon Fibre Reinforced ONYX		
Printer	Prusa CoreOneL	Markforged X7	Markforged X7		
Slicer	Prusa Slicer	Eiger	Eiger		
Layer Height	0.15 mm	0.125 mm	0.125 mm		
Nozzle Diameter	0.4 mm	0.4 mm	0.4 mm		
Nozzle temperature	290 C	275 C	275 C		
Bed temperature	50 C	—	—		
Number of walls	1	1	1		
Bottom layers	2	1	1		
Top Layers	2	1	1		
Infill	Gyroid 28%	Gyroid 28%	Gyroid 28%		
Fibre Stripe count	—	—	1 stripe per 6 mm height		
Layers per stripe	—	—	4		
Continuous fibre volume fraction	—	—	Compression	3-Point Bending	DMA
			~8% *	~9.3% *	~7.4% *

* The continuous carbon fibre percentages reported for the ONYX-R specimens represent the estimated volumetric fibre content provided by the slicing software. Variations between specimen geometries arise from differences in the generated reinforcement paths and the corresponding fibre volume. These values were not determined experimentally.

**Table 3:** Compression properties of the investigated sandwich configurations, expressed as mean ± standard deviation (SD).

Sandwich Configuration	Compression Strength (MPa)	Compression Modulus (MPa)	Yield Strength (0.2%) [MPa]
QFRP-PPA-CF *	15.90 ± 0.10	86.26 ± 0.42	11.70 ± 0.10
QFRP-ONYX-R	15.97 ± 0.05	76.24 ± 2.76	12.45 ± 0.16
QFRP-ONYX	15.99 ± 0.07	22.08 ± 2.69	2.00 ± 0.09

* For the QFRP-PPA-CF configuration, specimen 1 exhibited a substantially different compressive response compared with the other specimens and was excluded from the reported statistical analysis. Consequently, the mean values and standard deviations were calculated using specimens 2 and 3 (*n* = 2).

**Table 4:** Flexural properties of the investigated sandwich configurations.

Sandwich Configuration	Max. Flexural Stress [MPa]	Flexural Stress at Break [MPa]	Flex Strain at Break [%]	Energy at Break [J]	Modulus [MPa]
QFRP-PPA-CF	131.55 ± 3.50	125.49 ± 2.18	2.63 ± 0.10	3.18 ± 0.29	7319 ± 72
QFRP-ONYX-R	109.62 ± 3.00	108.76 ± 3.21	2.68 ± 0.20	2.75 ± 0.33	5977 ± 93
QFRP-ONYX	25.91 ± 1.59	19.62 ± 2.01	9.38 ± 1.02	3.13 ± 0.48	1483 ± 67

## Data Availability

The original contributions presented in this study are included in the article. Further inquiries can be directed to the corresponding author.
